# Community health workers adherence to referral guidelines: evidence from studies introducing RDTs in two malaria transmission settings in Uganda

**DOI:** 10.1186/s12936-016-1609-7

**Published:** 2016-11-24

**Authors:** Sham Lal, Richard Ndyomugenyi, Lucy Paintain, Neal D. Alexander, Kristian S. Hansen, Pascal Magnussen, Daniel Chandramohan, Siân E. Clarke

**Affiliations:** 1 Department of Disease Control, Faculty of Infectious Tropical Diseases, London School of Hygiene and Tropical Medicine, Keppel Street, London, UK; 2 C/O Vector Control Division, Ministry of Health, Kampala, Uganda; 3 MRC Tropical Epidemiology Group, Department of Infectious Disease Epidemiology, Faculty of Epidemiology and Population Health, London School of Hygiene and Tropical Medicine, London, UK; 4 Institute of Public Health, University of Copenhagen, Copenhagen, Denmark; 5 Faculty of Health and Medical Sciences, Institute of International Health, Immunology and Microbiology & Institute of Veterinary Disease Biology, University of Copenhagen, Copenhagen, Denmark

## Abstract

**Background:**

Many malaria-endemic countries have implemented national community health worker (CHW) programmes to serve remote populations that have poor access to malaria diagnosis and treatment. Despite mounting evidence of CHWs’ ability to adhere to malaria rapid diagnostic tests (RDTs) and treatment guidelines, there is limited evidence whether CHWs adhere to the referral guidelines and refer severely ill children for further management. In southwest Uganda, this study examined whether CHWs referred children according to training guidelines and described factors associated with adherence to the referral guideline.

**Methods:**

A secondary analysis was undertaken of data collected during two cluster-randomized trials conducted between January 2010 and July 2011, one in a moderate-to-high malaria transmission setting and the other in a low malaria transmission setting. All CHWs were trained to prescribe artemisinin-based combination therapy (ACT) and recognize symptoms in children that required immediate referral to the nearest health centre. Intervention arm CHWs had additional training on how to conduct an RDT; CHWs in the control arm used a presumptive diagnosis for malaria using clinical signs and symptoms. CHW treatment registers were reviewed to identify children eligible for referral according to training guidelines (temperature of ≥38.5 °C), to assess whether CHWs adhered to the guidelines and referred them. Factors associated with adherence were examined with logistic regression models.

**Results:**

CHWs failed to refer 58.8% of children eligible in the moderate-to-high transmission and 31.2% of children in the low transmission setting. CHWs using RDTs adhered to the referral guidelines more frequently than CHWs not using RDTs (moderate-to-high transmission: 50.1 vs 18.0%, p = 0.003; low transmission: 88.5 vs 44.1%, p < 0.001). In both settings, fewer than 20% of eligible children received pre-referral treatment with rectal artesunate. Children who were prescribed ACT were very unlikely to be referred in both settings (97.7 and 73.3% were not referred in the moderate-to-high and low transmission settings, respectively). In the moderate-to-high transmission setting, day and season of visit were also associated with the likelihood of adherence to the referral guidelines, but not in the low transmission setting.

**Conclusions:**

CHW adherence to referral guidelines was poor in both transmission settings. However, training CHWs to use RDT improved correct referral of children with a high fever compared to a presumptive diagnosis using sign and symptoms. As many countries scale up CHW programmes, routine monitoring of reported data should be examined carefully to assess whether CHWs adhere to referral guidelines and take remedial actions where required.

**Electronic supplementary material:**

The online version of this article (doi:10.1186/s12936-016-1609-7) contains supplementary material, which is available to authorized users.

## Background

In many sub-Saharan African countries where malaria is endemic, community health workers (CHWs) have received renewed interest to deliver primary healthcare in areas with poor access to public health services, and CHW programmes to treat common childhood infections of malaria, pneumonia and diarrhoea (known as integrated community case management (iCCM)) have been introduced in over 25 countries with the aim of reducing under-five mortality [1, 2]. Previous studies have shown that adequately trained CHWs can correctly diagnose and treat children with uncomplicated malaria, resulting in mortality reductions [3, 4]. Although CHW training usually includes guidance on when children should be referred to a fully qualified health worker, few studies have examined CHW adherence to these referral guidelines [3, 5–7]. Guidelines can serve to: (1) support CHWs to make appropriate referral decisions; (2) encourage caregivers to seek further care from health facilities; and, (3) ensure CHWs do not risk managing illnesses they are not trained for, and thus limit adverse outcomes that may arise if children do not receive attention from qualified health workers.

In contrast to the growing literature on CHW adherence to treatment guidelines, there is much less describing adherence to referral guidelines, and findings are mixed, with referral ranging from 9 to 83% [8–10]. Provision of guidelines to CHWs on which illnesses should be referred to health centres is the start of a complex process involving CHWs, caregivers and health workers in health facilities [11]. First, CHWs need to have the skills to identify and distinguish children with severe signs and symptoms from those who do not, and refer them promptly. Second, when referrals are made, caregivers need to adhere to the advice. Last, health facilities need to be prepared to receive, assess and treat referred cases promptly and effectively. The lack of evidence on referral has been highlighted as a priority for further research to inform the implementation and scale-up of iCCM globally [12–15].

Previous studies on adherence to referral were small-scale evaluations involving a few CHWs, conducted in the context of a presumptive clinical diagnosis for malaria, before WHO-recommended parasitological testing with malaria rapid diagnostics tests (RDTs) at all levels of the health system, including the community [16]. There is urgent need for contemporary data, based on larger samples, to be representative of the range of referral practices amongst CHWs. In this analysis, data collected during trials conducted in Uganda to evaluate the effect of a CHW intervention using RDTs on malaria treatment, was used to assess CHW adherence to referral guidelines [17]. These two trials provided an opportunity to describe CHW adherence to referral guidelines and explore the factors related to adherence when CHWs were trained to use RDTs and prescribe artemisinin-based combination therapy (ACT).

## Methods

### Study context

The two cluster-randomized trials were conducted in Rukungiri District, southwestern Uganda; one trial was conducted in villages in a moderate-to-high malaria transmission setting (Bwambara sub-county, 980–1200 m above sea level) and another was conducted in villages in a low transmission highland setting (Nyakishenyi sub-county, 1064–2157 m above sea level) [18]. More than 85% of the population in both settings lived in rural areas and the main occupation of Bahororo and Bakiga ethnic groups was subsistence agriculture [19]. The climate is characteristic of East African tropics with two rainy seasons, March–May and September–December, and annual temperatures ranging between 16 and 25 °C. Malaria transmission is perennial with peaks in incidence shortly after the rains. The public health system in each sub-county comprises three health centres, two classed as public health centre IIs (HCII) and one classed as health centre III (HCIII). HCIIs provide outpatient and community outreach services, whilst HCIIIs provide curative and preventative services and supervise lower level HCIIs; they also act as the first referral cover for the sub-county [20, 21].

**Fig. 1:**
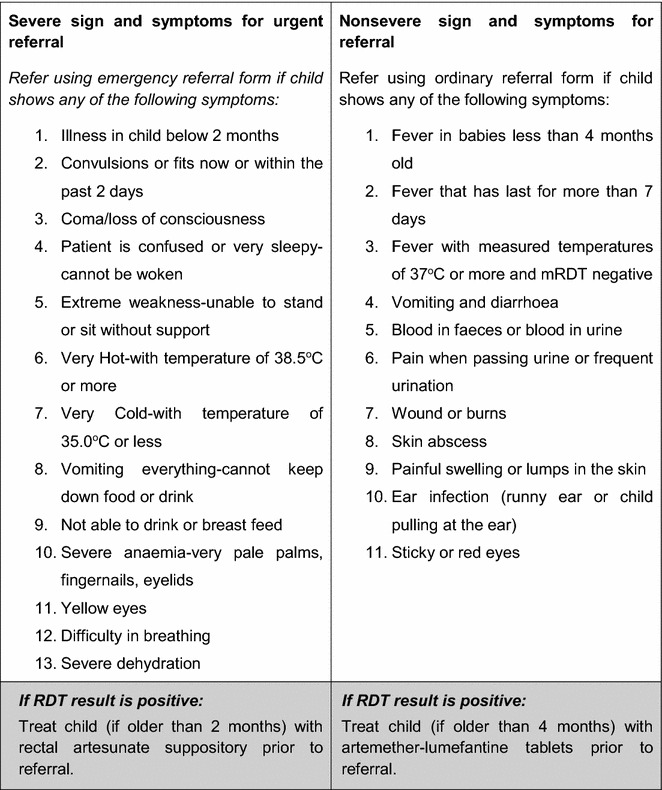
List of severe and non-severe signs and symptoms that community health workers (CHWs) were trained to identify and refer in children

Prior to the trials starting, community meetings were held to select CHWs for training and sensitize local communities on diagnostic testing for malaria. The key messages were: not all fevers are malaria and a diagnostic test was advisable before treatment by ACT (using artemether-lumefantrine); that a quick malaria test (RDT) could test for malaria, and, tests were available from CHWs in villages in the intervention arm. In January 2010, 381 CHWs (192 CHWs in moderate-to-high transmission setting, 189 CHWs in the low transmission setting) were trained to: (a) receive children presenting with fever and their caregivers; (b) take a history of a child’s symptoms and diagnose malaria; (c) treat a child with uncomplicated malaria; and, (d) record basic information, including treatment decisions and drugs prescribed. All CHWs were provided with digital thermometers and trained to measure axillary temperature in children with a history of fever. The training also covered the identification of signs and symptoms of other illnesses that CHWs were not trained to manage and that required referral to health centres for investigation. Severe signs and symptoms for immediate referral included: convulsions or fits, extreme weakness, coma loss of consciousness, and high temperature of 38.5 °C or more; whilst non-severe referral signs and symptoms included: wounds or burns, ear infections, sticky or red eyes, and vomiting and diarrhoea (Fig. 1). The danger signs for urgent referral were chosen to identify severe forms of malaria, cerebral malaria, meningitis, pneumonia, and/or severe bacterial infections. Other signs and symptoms for referral typically identified less serious illnesses that required management at a health centre, including gastro-intestinal infections, skin infections, otitis media, conjunctivitis, and/or respiratory tract infections. The referral criteria were based on the research team’s clinical experience and national treatment guidelines in Uganda at the time [22]. CHWs were trained to treat only malaria, had limited case management experience before this study, and the criteria for referral thus veered on the side of caution, aiming to ensure children with danger signs and/or non-malarial illnesses were treated at a health centre.

In addition to training how to identify signs and symptoms for referral, CHWs in the intervention arm of each trial (93 moderate-to-high transmission setting, 96 low transmission setting) received training on how to perform an RDT and prescribe an age-dependent oral dose of ACT after a positive RDT result. In contrast, RDT-negative children were not prescribed an ACT, and referred if the CHW identified any of the listed signs and symptoms for referral (Fig. 1). CHWs in the control arm were trained to prescribe an ACT based on a presumptive diagnosis of malaria if a child had an axillary temperature >37.5 °C. CHWs in both arms were trained to administer rectal artesunate if a child presented with one or more severe signs and symptoms, including high fever (temperature of ≥38.5 °C) and to refer the child to the nearest health centre for further management [23]. The job aids summarizing the decisions CHWs were trained to make in each arm are shown in Additional file 1: Figure S1.

The CHWs began treating children in May 2010 in the moderate-to-high transmission setting and in June 2010 in the low transmission setting. CHW training was reinforced through close-support supervision for the first few months, with weekly visits by a field coordinator to review and collect CHW treatment records, referral forms and stock cards, and to discuss concerns or difficulties of carrying out their roles. From January 2011, supervision of CHWs was scaled back and limited to monthly meetings to reflect typical levels of supervision under programmatic conditions.

The aim of both trials was to evaluate the effectiveness of training CHWs to use RDTs to diagnose and treat malaria with ACT compared with CHWs using a presumptive diagnosis for the management of malaria. The primary trial endpoint was the proportion of febrile children with malaria receiving appropriately targeted treatment with an ACT [17]. In this secondary analysis, an assessment of whether CHWs adhered to the referral guidance and decided to refer children, by examining adherence to one of the criteria for urgent referral: high fever with an axillary temperature ≥38.5 °C.

### Data analyses

**Fig. 2:**
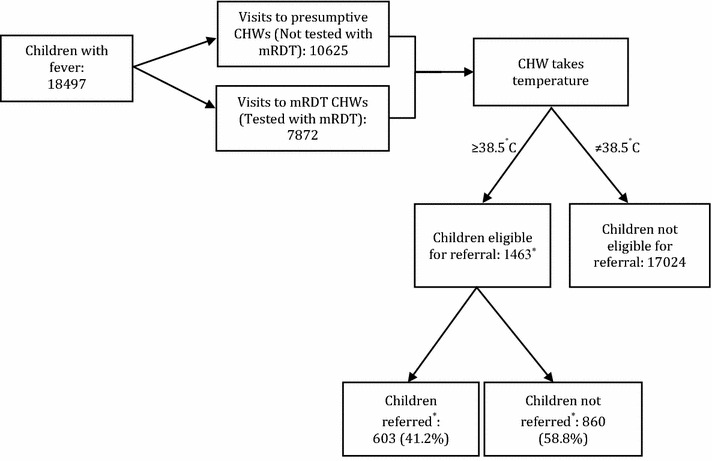
Profile of children analysed in the moderate-to-high transmission setting. *Referral outcome missing for ten children

The analysis examined whether CHWs in each transmission setting adhered to referral guidance in children who presented with a high fever (temperature ≥38.5 °C); this indicator of adherence to referral guidelines was selected because axillary temperature was the only sign routinely recorded by CHWs for all children. Therefore, the recorded temperature was used to identify children who should, irrespective of the RDT result, have been referred according to the training guidelines and examine whether these children were actually referred by CHWs (Fig. 2). The study did not have an independent assessment of the other 11 severe signs and symptoms for referral. Data was analysed from the treatment registers completed by CHWs between January 2011 and July 2012, after CHW supervision was scaled back.

All data were double entered and verified using Microsoft Access 2007 (Microsoft Corp, Redmond, WA, USA); village distances to the nearest health centre were calculated using ArcGIS Desktop 10.3 (Environmental Systems Research Institute Inc, Redlands, CA, USA). All data were analysed using STATA version 14.1 (STATA Corp, College Station, TX, USA).

The outcome for this analysis was CHW adherence to the referral guideline, defined as the proportion of children presenting with high fever (temperature ≥38.5 °C) that were referred by CHWs. An exploratory analysis of each trial’s dataset was undertaken to identify factors associated with adherence to referral guideline in which child visits to CHW were grouped into three categories: (1) children who visited CHWs using a presumptive diagnosis for malaria (without RDT); (2) children who tested RDT positive; and, (3) children who tested RDT negative. Additional factors routinely recorded in CHW treatment registers which were considered potentially affecting CHW adherence to referral guidelines included: child’s age, gender, duration of fever, and the use of an insecticide-treated net (ITN) the previous night. Time of visit (weekday or weekend) was derived from the date of the child’s visit, and rainy season visits were defined as visits in March–May and September–December. Euclidean (straight-line) distance was estimated from the centre of a village to the nearest health centre.

For each transmission setting, an explanatory model for the outcome of referral was developed using logistic regression; odds ratios (OR) and 95% confidence intervals (95% CI) were calculated with random effects to account for clustering at the village level [24]. An unadjusted analysis was used to identify factors associated with CHW referral of children with a high fever. To examine CHW adherence to the referral guidelines and identify independent factors associated with referral, all factors identified a priori were included in the adjusted model. In both the unadjusted and adjusted models factors associated with referral were assessed using a log-likelihood ratio test.

## Results

### Child characteristics

**Fig. 3:**
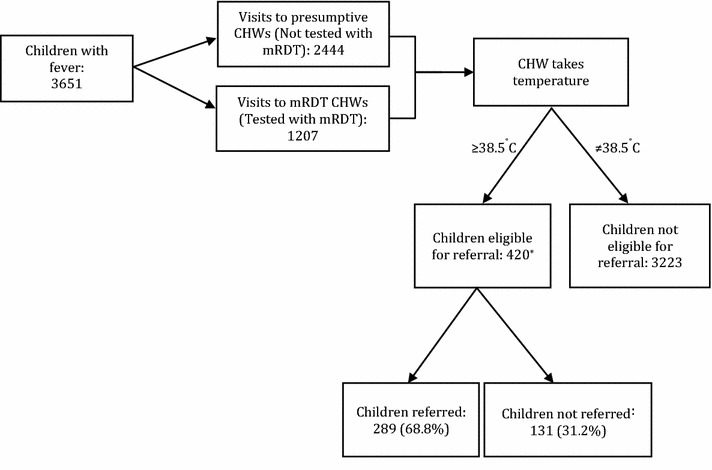
Profile of children analysed in the low transmission setting. *Referral outcome missing for eight children

During the 19-month study period (January 2011–July 2012), 18,497 children with a history of fever were seen by 180 CHWs in the moderate-to-high transmission setting, of whom 8.0% (1473/18,497) were eligible for referral with a high fever (temperature ≥38.5 °C) according to the referral guidelines (Fig. 2). In the low transmission setting, 13.3% (428/3223) of children visiting the 189 CHWs had a temperature ≥38.5 °C (Fig. 3). The characteristics of children with high fever were broadly similar in both transmission settings: nearly half were aged between 1 and 3 years and similar proportions were males and females (Table 1). A large proportion of caregivers reported their child had slept under a net the previous night, and >80% had sought care within 24 h of onset of fever. Most children were seen on a weekday, with more visits occurring during the two rainy seasons compared to the dry seasons (Table 1). There were a few differences between the two transmission settings: a higher proportion of children were tested with an RDT in the moderate-to-high transmission setting compared to the low transmission setting (72 and 55%, respectively), and villages in the low transmission setting were located further away from the nearest public health centre than villages in moderate-to-high transmission setting (Table 1).

**Table 1:** Characteristics of children who visited CHWs and were eligible for referral (presented with a temperature ≥38.5 °C)

	Moderate-to-high transmission setting (%)^a^	Low transmission setting (%)^b^
	N = 1473	N = 428
Age group (years)		
<1.0	296 (20.2)	116 (27.4)
1.0–2.9	630 (43.0)	179 (42.3)
3.0–4.9	530 (36.2)	128 (30.3)
5.0–15.0	8 (0.5)	0 (0.0)
Sex		
Male	761 (52.0)	209 (48.8)
Female	703 (48.0)	219 (51.2)
Slept under a net the previous night		
No	141 (9.7)	50 (11.9)
Yes	1312 (90.3)	371 (88.1)
Resident in the same village as a CHW		
No	123 (8.4)	73 (17.1)
Yes	1345 (91.6)	355 (82.9)
Time of visit to CHW after onset of symptoms (h)		
>24	181 (12.5)	65 (15.6)
Within 24	1266 (87.5)	352 (84.4)
Tested with RDT		
Not tested	406 (27.6)	192 (44.9)
Tested	1067 (72.4)	236 (55.1)
Day of visit to a CHW		
Weekday	1054 (71.6)	312 (72.9)
Weekend	419 (28.4)	116 (27.1)
Season of visit to a CHW		
Dry	512 (34.8)	162 (37.9)
Wet	961 (65.2)	266 (62.1)
Village distance to nearest health centre (km)		
0.0–2.4	690 (47.0)	97 (24.0)
2.5–4.9	727 (49.5)	159 (39.3)
5.0–7.4	51 (3.5)	103 (25.4)
7.5–8.9	0 (0.0)	46 (11.4)

^a^Data missing in the moderate-to-high transmission setting, for age: 9; gender: 9; net use: 20; resident in the same village: 5; onset of symptoms 26

^b^Data missing in the low transmission setting, for age: 5; gender: 0; net use: 7; resident in the same village: 0; onset of symptoms 11

### CHW adherence to referral guideline

In both transmission settings, CHWs did not always adhere to the guidelines and refer all children that were eligible for referral (temperature ≥38.5 °C). In the moderate-to-high transmission setting, CHWs failed to refer 58.8% (860/1463) of eligible children; this was higher than the low transmission setting, where 30.6%, 131/420 of eligible children were not referred (Figs. 2, 3). Table 2 shows the proportion of eligible children who were referred, categorized by whether CHWs tested with an RDT or not, their RDT result and whether an ACT was prescribed. In both settings, CHWs adhered to the referral guideline more often when children were tested with an RDT compared to those who were not tested (moderate-to-high transmission: 50.1 vs 18.0%, p = 0.003, Table 2a; low transmission: 88.5 vs 44.1%, p < 0.001, Table 2b). In both settings, CHWs also adhered to the referral guideline more frequently when children were RDT negative as opposed to positive. The frequency of CHW adherence to the referral guideline was generally higher among children seen in the low transmission setting than among children seen in moderate-to-high transmission setting, across all categories (tested/untested; RDT-positive/RDT-negative; ACT prescribed/not prescribed).

**Table 2:** CHWs referral practices amongst eligible children (temperature ≥38.5 °C) by RDT result and ACT prescription in each transmission setting

(a) Moderate-to-high transmission setting	Total	Referred (%)	Not referred (%)
Overall^a^	1463	603 (41.2)	860 (58.8)
RDT			
Not tested	405	73 (18.0)	332 (82.0)
Tested	1058	530 (50.1)	528 (49.9)
Within those tested with RDT			
RDT Negative	657	497 (75.6)	160 (24.4)
RDT Positive	401	33 (8.2)	368 (91.8)
ACT prescription^b^			
ACT not prescribed	665	504 (75.8)	161 (24.2)
ACT prescribed	709	16 (2.3)	693 (97.7)
Rectal artesunate prescribed	47	47 (100.0)	0 (0.0)

(b) Low transmission setting			
Overall^c^	420	289 (68.8)	131 (31.2)
RDT			
Not tested	186	82 (44.1)	104 (55.9)
Tested	234	207 (88.5)	27 (11.5)
Within those tested with an RDT			
RDT negative	225	202 (89.8)	23 (10.2)
RDT positive	9	5 (55.6)	4 (44.4)
ACT prescription^d^			
ACT not prescribed	225	201 (89.3)	24 (10.7)
ACT prescribed	146	39 (26.7)	107 (73.3)
Rectal artesunate prescribed	37	37 (100.0)	0 (0.0)

^a^10 missing referral status

^b^42 missing treatment prescription

^c^8 missing referral status

^d^12 missing treatment prescription

Referral of eligible children also varied by whether CHWs prescribed ACT and was less frequent when ACT was prescribed. In the moderate-to-high transmission setting only 2.3% of eligible children prescribed an ACT were referred, while 75.8% of children not prescribed an ACT were referred (Table 2a). In the low transmission setting, 26.7% of children who were prescribed an ACT were referred whilst 89.3% who were not prescribed ACT were referred (Table 2b). Use of rectal artesunate was generally low; in total 47 and 37 children with high fever in the moderate-to-high transmission and low transmission setting, respectively, received rectal artesunate during the 19-month trial, all of whom were subsequently referred according to the guideline. CHWs not testing with an RDT rarely prescribed rectal artesunate: only 7.2% (27/374) and 16.6% (27/163) of children with high fever not tested received rectal artesunate in the moderate-to-high transmission and low transmission sites, respectively. Similarly, CHWs using RDT gave rectal artesunate in only 4.3% (17/397) of RDT-positive children with high fever in the moderate-to-high transmission setting, and in 11.1% (1/9) of eligible children in the low transmission setting.

### Factors associated with CHW adherence to referral guideline: moderate-to-high transmission setting

CHWs in the moderate-to-high transmission setting were less likely to adhere to the referral guideline (referring all children with a temperature ≥38.5 °C) when ACT was prescribed (adjusted OR (AOR) 0.0025; 95% CI 0.00061–0.0099; p < 0.001); when a child visit occurred on the weekend compared to a weekday (AOR 0.62; 95% CI 0.41–0.95; p = 0.027), and during the wet season compared to the dry season (AOR 0.65; 95% CI 0.42–0.99; p = 0.043) (Table 3). After controlling for other variables, the adjusted analysis found no association between the likelihood of CHW adherence to referral guideline and the child’s age or RDT result.

**Table 3:** Factors associated with CHW adherence to referral guideline among children eligible for referral (temperature ≥38.5 °C) in the moderate-to-high transmission setting

Variables	Eligible for referral (≥38.5 °C)	Referrals made (%)	Unadjusted odds ratio (95% CI)	p value	Adjusted odds ratio (95% CI)	p value
Test result						
Not tested	405	73 (18.0)	1		1	
RDT negative	657	497 (75.6)	6.93 (3.45–13.92)	<0.001	0.54 (0.16–1.79)	0.567
RDT positive	401	33 (8.2)	0.11 (0.05–0.24)		1.03 (0.29–3.61)	
Age group (years)						
<1.0	292	155 (53.1)	1		1	
1.0–2.9	628	258 (41.1)	0.55 (0.39–0.78)		0.98 (0.59–1.63)	
3.0–4.9	527	184 (34.9)	0.39 (0.27–0.56)	<0.001	1.11 (0.64–1.91)	0.805
5.0–15.0	8	3 (37.5)	0.38 (0.07–1.98)		0.45 (0.64–1.91)	
Gender						
Male	756	306 (40.5)	1		1	
Female	698	290 (41.5)	0.94 (0.73–1.21)	0.626	1.05 (0.71–1.56)	0.795
Slept under a net the previous night						
No	138	65 (47.1)	1		1	
Yes	1305	523 (40.1)	1.35 (0.84–2.17)	0.211	1.39 (0.66–2.92)	0.382
Resident in the same village as a CHW						
No	122	73 (59.8)	1		1	
Yes	1336	526 (39.4)	0.73 (0.46–1.16)	0.184	0.59 (0.28–1.25)	0.169
Time of visit to CHW after onset of symptoms (h)						
>24	181	90 (49.7)	1		1	
Within 24	1256	496 (39.5)	0.77 (0.53–1.13)	0.189	0.70 (0.39–1.27)	0.244
ACT prescription						
No ACT	665	504 (75.8)	1		1	
ACT	709	16 (2.3)	0.004 (0.002–0.009)	<0.001	0.003 (0.001–0.010)	<0.001
Day of visit to a CHW						
Weekday	1046	451 (43.1)	1		1	
Weekend	417	152 (36.5)	0.74 (0.56–0.98)	0.033	0.62 (0.41–0.95)	0.027
Season of visit to a CHW						
Dry	508	235 (46.3)	1	0.004	1	
Wet	955	368 (38.5)	0.68 (0.52–0.88)		0.65 (0.42–0.99)	0.043
Village distance to nearest health centre (km)						
0.0–2.4	686	337 (49.1)	1	0.093	1	0.060
2.5–4.9	721	251 (34.8)	0.52 (0.28–0.97)		0.39 (0.18–0.86)	
5.0–7.4	51	14 (27.5)	0.41 (0.08–2.12)		0.93 (0.12–7.07)	
7.5–8.9	N/A	N/A	N/A		N/A	

### Factors associated with CHW adherence to referral guideline: low transmission setting

In the low transmission setting the adjusted analysis found that CHWs were more than seven times more likely to adhere to the referral guideline and refer RDT-negative children, compared to those not tested (OR 7.14; 95% CI 1.99–25.59; p = 0.010; Table 4); and more than three times more likely to adhere when children tested positive (OR 3.19; 95% CI 0.38–26.87; p = 0.010). There was also an association with malaria treatment, independent of RDT result: CHWs were less likely to adhere to the referral guideline if they prescribed an ACT compared to not prescribing an ACT (OR 0.07; 95% CI 0.02–0.26; p < 0.001). Unlike the moderate-to-high transmission setting, there were no associations between adherence to the referral guideline and distance to the nearest health centre, day or season of the visit.

**Table 4:** Factors associated with CHW adherence to referral guideline among children eligible for referral (temperature ≥38.5 °C) in the low transmission setting

Variables	Eligible for referral (≥38.5 °C)	Referrals made (%)	Unadjusted odds ratio (95% CI)	p value	Adjusted odds ratio (95% CI)	p value
Test result						
Not tested	186	82 (44.1)	1		1	
RDT negative	225	202 (89.8)	17.00 (6.53–44.26)	<0.001	7.14 (1.99–25.59)	0.010
RDT positive	9	5 (55.6)	1.23 (0.20–7.53)		3.19 (0.38–26.87)	
Age group (years)						
<1.0	113	82 (72.6)	1		1	
1.0–2.9	174	128 (73.6)	0.95 (0.46–1.96)		1.47 (0.59–3.69)	
3.0–4.9	128	76 (59.4)	0.55 (0.26–1.16)	0.195	0.64 (0.25–1.63)	0.158
5.0–15.0	0	0 (0.0)			1.00 (0.25–1.63)	
Gender						
Male	203	137 (67.5)	1		1	
Female	217	152 (70.0)	1.26 (0.70–2.27)	0.436	1.63 (0.78–3.42)	0.193
Slept under a net the previous night						
No	50	38 (76.0)	1		1	
Yes	363	244 (67.2)	0.69 (0.29–1.64)	0.398	1.70 (0.51–5.64)	0.389
Resident in the same village as a CHW						
No	72	49 (68.1)	1		1	
Yes	348	240 (69.0)	1.89 (0.83–4.31)	0.131	3.05 (0.94–9.92)	0.064
Time of visit to CHW after onset of symptoms (h)						
>24	63	51 (81.0)	1		1	
Within 24	346	232 (67.1)	0.44 (0.18–1.07)	0.072	0.67 (0.23–1.89)	0.445
ACT prescription						
No ACT	225	201 (89.3)	1		1	
ACT	146	39 (26.7)	0.03 (0.01–0.07)	<0.001	0.07 (0.02–0.26)	<0.001
Day of visit to a CHW						
Weekday	307	213 (69.4)	1		1	
Weekend	113	76 (67.3)	1.42 (0.74–2.75)	0.295	1.21 (0.53–2.74)	0.649
Season of visit to a CHW						
Dry	158	96 (60.8)	1	0.008	1	
Wet	262	193 (73.7)	2.24 (1.24–4.06)		1.67 (0.77–3.60)	0.191
Village distance to nearest health centre (km)						
0.0–2.4	96	61 (63.5)	1		1	
2.5–4.9	158	116 (73.4)	1.29 (0.27–6.29)		0.78 (0.22–2.79)	
5.0–7.4	100	63 (63.0)	1.41 (0.27–7.41)	0.846	0.55 (0.14–2.14)	0.200
7.5–8.9	43	34 (79.1)	3.11 (0.27–7.41)		5.41 (0.68–42.80)	

### Other symptoms in children referred for high fever

A supplementary analysis was undertaken to describe the other signs and symptoms recorded by CHWs amongst children referred with a high fever (temperature ≥38.5 °C). Nearly all CHWs who referred children with a high fever correctly reported this as a symptom for referral on the form for urgent referrals (moderate-to-high transmission setting, 51/54, Additional file 1: Table S1; low transmission setting, 22/23, Additional file 1: Table S2). Among severe signs and symptoms for referral, the most frequently reported were *“difficulty in breathing”* and “*not able to drink or breastfeed”*, in both settings (Additional file 1: Tables S1, S2). Less frequent severe signs and symptoms included convulsions and extreme weakness. Although all children should have been given a severe referral form because they had a high fever, CHWs also referred children using the form for non-severe signs and symptoms, most commonly for “*fever with measured temperature of* >*37* *°C and RDT negative*”. Other symptoms, included *“fever that had lasted for seven days”*, “*vomiting and diarrhoea*”, “*pain when passing urine*”, and “*wounds or burns”*. In the moderate-to-high transmission setting, high fever was reported as the exclusive sign for referral in 29.6% (16/54) of referrals, and alongside other severe signs and symptoms in 70.4% (38/54) of all severe referral forms (Additional file 1: Table S1). In the low transmission setting, CHWs reported high fever exclusively on 6 of the 23 severe referral forms, and alongside other severe signs and symptoms on 17 forms (Additional file 1: Table S2).

In both transmission settings, there was a discrepancy between the number of referrals recorded in the CHW treatment register and the number of referral forms (used to analyse the reasons for referral, summarized above). In the moderate-to-high transmission setting, 603 referrals were recorded but referral forms were only available for 74 (12.3%) of these. In the low transmission setting, 420 referrals were recorded but only 34 (8.1%) referral forms were available.

## Discussion

These results suggest that CHWs failed to refer up to 60% of children who should have been referred according to the referral guideline (high fever temperature ≥38.5 °C). However, this study also found that CHWs using RDTs in the low transmission setting adhered more frequently to referral guidance compared to CHWs using presumptive diagnosis based solely on signs and symptoms; both RDT-negative and -positive children were more likely to be referred compared to CHWs not testing with an RDT. CHW adherence to ACT treatment guidelines was no better: all children with a high fever who were RDT-positive (or not tested with an RDT) should have received rectal artesunate pre-referral treatment, but fewer than 20% of these children received the correct treatment. Failure to prescribe pre-referral treatment (rectal artesunate) and refer is a concern because not referring these children for further management has the potential to worsen their condition.

Children with high fevers not referred were often RDT-positive and prescribed an ACT, suggesting CHWs may have overlooked the need for referral once an ACT was given. In contrast, RDT-negative children not prescribed ACT were more likely to be referred to the nearest health centre for further management. These results also show poor CHW adherence to rectal artesunate prescribing guidelines; the WHO guidance for the management of severe malaria in primary care recommends pre-referral treatment with rectal artesunate and referral [25]. Children with high fevers who visited CHWs using a presumptive diagnosis and those who were RDT positive should all have received rectal artesunate and been referred according to the study guidelines, due to their increased risk of severe malaria (Additional file 1: Figure S1). However, in both settings children with a high fever who were RDT positive or not tested were frequently prescribed an ACT and not referred. Despite the low use of rectal artesunate overall, it is reassuring that when CHWs did prescribe rectal artesunate, they always referred.

In addition to malaria test result and treatment, the CHWs’ decision to refer also differed according to both the day and season of visit, in one study area. This could possibly be due to CHWs knowing that local health centres (HCII and III) would be closed during the weekend and that access would be difficult due to the poor condition of roads during the rainy season, factors which might affect whether the caregiver would take their child to a health centre. In contrast, in the other transmission setting there was no evidence of an association between the day or season of visit and referral. This difference could have been due to the existence of a nearby mission-run hospital open during the weekend, enabling CHWs to refer in the belief that caregivers would be likely to seek further care. There is also the possibility that CHWs did not refer because caregivers influenced the decision to refer. Although this was not captured systematically, ad-hoc evidence from the open-text comments section of the CHW treatment record forms confirm instances where caregivers refused referral and CHWs did not refer them (3% of all comments made). Similar observations were made by Winch et al. who found CHWs were more likely to make a referral when they knew caregivers would comply with their advice [26].

Adherence to the referral guidelines by CHWs was generally higher in the low transmission setting compared to the moderate-to-high transmission setting. This may be due to differences between the two trial sites. First, CHWs in the low transmission setting saw fewer children than CHWs in the moderate-to-high transmission setting, and may have been less trusting of the malaria test and/or their competence in deciding when to refer. CHWs who are less confident in their role may prefer to refer according to the guidelines and not risk further complications in the child. In contrast, CHWs in the moderate-to-high transmission experienced higher number of visits, will have obtained case management experience more rapidly, and may have made their own judgements on when to prescribe rectal artesunate or refer. Similar patterns have been reported in other community-based studies in Uganda, where drug shop vendors became increasingly confident in their skills to manage clients and were reluctant to refer clients [27]. Second, the low transmission setting is historically prone to epidemics, children develop acquired immunity more slowly due to reduced malaria exposure, and may be more likely to develop severe malaria if not treated [28, 29]. CHWs may have been aware of this risk and more inclined to refer. Finally, the presence of a nearby mission-run hospital with an insurance scheme in the low transmission setting may have enabled CHWs to refer knowing care would be available; no such hospital or insurance scheme was present in the moderate-to-high transmission setting.

In this analysis, adherence to referral guidelines was assessed through review of treatment records kept by CHWs; temperature was as an indicator of whether a referral should have been made because it was recorded for all children. There are potential limitations to this approach. First, direct observation with re-examination by a medical professional is an established method to assess health worker adherence to case management guidelines in hospitals [30, 31]. This analysis was limited to data available from the two trials, which did not use this method of evaluation. However, prior studies which have used direct observation to evaluate CHW performance have found evidence of the Hawthorne effect, where CHWs may have followed guidelines more accurately under observation in a clinical setting compared to their community environment [8, 32]. Register review has previously been found to approximate results from a medical professional directly observing CHW performance [31, 32], and can offer a number of advantages: we were able to screen and analyse approximately 22,000 records in less time, with fewer resources compared to a direct observation method, without having to remove CHWs or health centre workers from their normal work. Second, the analysis was limited to data that were routinely recorded, but other unrecorded factors could also have influenced a CHW’s decision to refer or not, including perceptions of the availability and quality of care available at local facilities, caregiver demands, and interpersonal relationships. However, since CHW supplies were regularly monitored as part of the trial, it cannot be certain that non-adherence to the guideline was not due to stock-outs of rectal artesunate or referral forms. Finally, the single criterion (temperature ≥38.5 °C) used in this analysis is not necessarily representative of all criteria for referral, of all children needing referral, or adherence to referral guidelines for other symptoms. Indeed, when children with high fever were referred CHWs often reported additional severe signs and symptoms for referral alongside the temperature criterion, suggesting that CHWs may have thought that high fever on its own did not justify a referral. It is conceivable that other severe signs and symptoms may have had higher referral rates; also, that referral might be lower for non-severe signs and symptoms.

Despite these limitations the findings are comparable with two earlier studies in Ghana where CHWs used a presumptive malaria diagnosis, which found similar patterns of low referral [9, 10]. In one, 5 out of 17 children with signs of severe disease requiring referral were referred with a form; the authors thought this might reflect CHWs’ limited confidence in their diagnosis and a preference to re-assess later the need for referral [9]. It is possible in this study that CHWs may have also followed up and reassessed severely ill children for referral, however this was not systematically recorded.

This is one of the first studies to investigate CHW adherence to referral guidelines when RDTs have been implemented as part of a community case management programme, and although CHWs in these trials were trained only to treat malaria, the findings may also have relevance for referral guidance within iCCM programmes. The use of RDTs in any programme will identify some children that a CHW is not equipped to treat, and guidance for managing RDT-negative children is needed. In both settings CHWs referred eligible children more frequently when they were RDT-negative compared to when they were RDT-positive or not RDT tested. Nonetheless, it is probably impractical that all children who test RDT-negative should be referred without consideration of other signs or symptoms. CHWs in this study were trained to identify more than 20 signs and symptoms for referral. Is this too many? Can inclusion of non-severe signs diminish the perceived importance of adherence to referral guidelines? More worrying, there was strong evidence that children treated by a CHW were not referred. Treatment for one condition can thus increase the risk that CHWs overlook other signs and symptoms requiring referral, underlining the emphasis that needs to be placed on the referral criteria during CHW training. Although children with a high fever are at increased risk of febrile convulsions, it is possible that, in the absence of other symptoms, high fever was not perceived by CHW or caregiver to be serious enough to justify referral. Caregivers may prefer to observe the progression of their child’s fever at home and may not comply with CHW referral advice to visit a health centre. Poor compliance to referral by caregivers has been reported in studies in Uganda and Sierra Leone [33, 34]. There is considerable heterogeneity in national and iCCM guidelines on the referral criteria used in the countries that are currently scaling-up iCCM [35]. Some, but not all, include high fever as a criterion for referral. Although the findings presented here are not necessarily generalizable to other referral criteria, the results highlight the need to understand how CHWs make decisions on when to refer, and the factors that influence these decisions. Failure to refer may result in delayed treatment seeking and poorer child health, which would partly undo the benefits of increasing access to primary healthcare afforded by using CHWs [36, 37].

## Conclusion

In this study, CHWs tended not to refer children presenting with high fever (temperature ≥38.5 °C) if they had confirmed malaria diagnosis with RDT and prescribed an ACT. This practice was inconsistent with the CHW training guidelines that recommend referring children presenting with one or more severe symptoms. In other settings where CHW interventions are being implemented, further research is required to fully understand when and how CHWs decide to refer children, and the factors that influence their decision, in order to refine the guidelines and improve management of febrile illness in children.

## Additional file

**Additional file 1.** Supplementary material, Figure S1, Tables S1 and S2.

## Abbreviations

ACT: artemisinin-based combination therapy
CHW: community health worker
HCII: Health Centre II
HCIII: Health Centre III
iCCM: integrated community case management
ITN: insecticide treated net
RDTs: malaria rapid diagnostic tests
95% CI: 95% confidence intervals
OR: odds ratio
AOR: adjusted odds ratio
