# Supplementary material for: Community health workers adherence to referral guidelines: evidence from studies introducing RDTs in two malaria transmission settings in Uganda
Source: Malar J. 2016 Nov 24;15:568. doi: 10.1186/s12936-016-1609-7 (PMC5121932; doi:10.1186/s12936-016-1609-7)
Supplement: Supplementary file 1 — Additional file 1. Supplementary material, Figure S1, Tables S1 and S2. [file 12936_2016_1609_MOESM1_ESM.docx]

**Supplementary Information**

| **Table S1 Signs and symptoms for referral amongst children eligible for referral (temperature ≥38.5°C) and referred in the moderate-to-high transmission setting** | | | |  |
| --- | --- | --- | --- | --- |
|  | Moderate-to-high transmission setting^a^ | | |  |
|  | **Not tested with an RDT** | **RDT negative** | **RDT positive** |  |
|  | Frequency (%) | Frequency (%) | Frequency (%) |  |
| ***Non-severe signs and symptoms for referral*** |  |  |  |  |
| Fever in babies less than 4 months old | 0 (0.0) | 0 (0.0) | 0 (0.0) |  |
| Fever that has lasted more than 7 days | 0 (0.0) | 3 (4.5) | 0 (0.0) |  |
| Fever with measured temperature of >37°C and RDT negative | 0 (0.0) | 42 (63.6) | 0 (0.0) |  |
| Vomiting and diarrhoea | 0 (0.0) | 4 (6.1) | 1 (33.3) |  |
| Blood in faeces or urine | 0 (0.0) | 0 (0.0) | 0 (0.0) |  |
| Pain when passing urine, or frequent urination | 0 (0.0) | 2 (3.0) | 0 (0.0) |  |
| Wounds or burns | 0 (0.0) | 2 (3.0) | 0 (0.0) |  |
| Skin abscess | 0 (0.0) | 1 (1.5) | 0 (0.0) |  |
| Painful swellings or lumps in the skin | 0 (0.0) | 1 (1.5) | 0 (0.0) |  |
| Ear infection (runny ear or child pulling at ear) | 0 (0.0) | 1 (1.5) | 0 (0.0) |  |
| Sticky or red eyes | 0 (0.0) | 3 (4.5) | 1 (33.3) |  |
| Other non-severe signs and symptoms (free text)^b^ | 0 (0.0) | 7 (10.6) | 1 (33.3) |  |
| **Total number of non-severe signs and symptoms reported** | **0** | **66** | **3** |  |
| Total number of non-severe referrals forms | 0 | 17 | 3 |  |
| Mean number of signs and symptoms reported per non-severe referral form | 0.0 | 3.9 | 1.0 |  |
| ***Severe signs for referral*** |  |  |  |  |
| Illness in child under 2 months | 0 (0.0) | 0 (0.0) | 0 (0.0) |  |
| Convulsions or fits now or within the past 2 days | 0 (0.0) | 4 (3.8) | 2 (16.7) |  |
| Coma/Loss of consciousness | 0 (0.0) | 2 (1.9) | 0 (0.0) |  |
| Patient is confused or very sleepy - cannot be woken | 1 (11.1) | 0 (0.0) | 0 (0.0) |  |
| Extreme weakness unable to stand or sit without support | 1 (11.1) | 4 (3.8) | 1 (8.3) |  |
| Very hot with temperature of 38.5°C or more | 3 (33.3) | 45 (42.5) | 3 (25.0) |  |
| Very cold with temperature of 35.0°C or less | 0 (0.0) | 0 (0.0) | 0 (0.0) |  |
| Vomiting everything-cannot keep down food or drink | 0 (0.0) | 5 (4.7) | 1 (8.3) |  |
| Not able to drink or breastfeed | 0 (0.0) | 16 (15.1) | 2 (16.7) |  |
| Severe anaemia very pale palms, fingernails, eyelids | 0 (0.0) | 2 (1.9) | 0 (0.0) |  |
| Yellow eyes | 0 (0.0) | 2 (1.9) | 1 (8.3) |  |
| Difficulty in breathing | 3 (33.3) | 19 (17.9) | 2 (16.7) |  |
| Severe dehydration | 0 (0.0) | 0 (0.0) | 0 (0.0) |  |
| Other severe signs and symptoms (free text)^c^ | 1 (11.1) | 7 (6.6) | 0 (0.0) |  |
| **Total number of severe signs and symptoms reported** | **9** | **106** | **12** |  |
| Total number of severe referral forms | 3 | 47 | 4 |  |
| Mean number of signs and symptoms reported per severe referral form | 3.0 | 2.3 | 3.0 |  |
|  |  |  |  |  |
| Total number of severe and non-severe signs and symptoms reported | 9 | 172 | 15 |  |
| Total non-severe and severe referral forms^d^ | 3 | 64 | 7 |  |
| Total referrals made | 73 | 497 | 33 |  |
| ^a^ Percentages are calculated based on the total number of signs and symptoms reported.  ^b^ Other non-severe signs and symptoms for referral included: RDT negative; cough and flu (5), diarrhoea (1), dysentery (1); not tested; cough flu (1).  ^c^ Other severe signs and symptoms included: RDT negative; very high temperature but RDT negative (5), cough flu, RDT negative (1); worms (1); RDT positive; very high temperature (1).  ^d^ 529 referral forms were missing when a referral was made (70 not tested, 433 RDT negative and 26 RDT positive) | | | |  |

| **Table S2 Signs and symptoms for referral amongst children eligible for referral (temperature ≥38.5°C) and referred in the low transmission setting** | | | | |  |
| --- | --- | --- | --- | --- | --- |
|  | **Low transmission setting^a^** | | |  |  |
|  | **Not tested with an RDT** | **RDT negative** | **RDT positive** | | |
|  | Frequency (%) | Frequency (%) | Frequency (%) | | |
| ***Non-severe signs and symptoms for referral*** |  |  |  | | |
| Fever in babies under 4 months old | 0 (0.0) | 0 (0.0) | 0 (0.0) | | |
| Fever that has lasted more than 7 days | 0 (0.0) | 2 (6.5) | 0 (0.0) | | |
| Fever with measured temperature of >37°C and RDT-negative | 0 (0.0) | 16 (51.6) | 0 (0.0) | | |
| Vomiting and diarrhoea | 0 (0.0) | 2 (6.5) | 0 (0.0) | | |
| Blood in faeces or urine | 0 (0.0) | 0 (0.0) | 0 (0.0) | | |
| Pain when passing urine, or frequent urination | 0 (0.0) | 2 (6.5) | 0 (0.0) | | |
| Wounds or burns | 0 (0.0) | 1 (3.2) | 0 (0.0) | | |
| Skin abscess | 0 (0.0) | 0 (0.0) | 0 (0.0) | | |
| Painful swellings or lumps in the skin | 0 (0.0) | 0 (0.0) | 0 (0.0) | | |
| Ear infection (runny ear or child pulling at ear) | 0 (0.0) | 0 (0.0) | 0 (0.0) | | |
| Sticky or red eyes | 0 (0.0) | 1 (3.2) | 0 (0.0) | | |
| Other non-severe signs and symptoms (free text)^b^ | 0 (0.0) | 7 (22.6) | 1 (0.0) | | |
| **Total number of non-severe signs and symptoms reported** | **0** | **31** | **0** | | |
| Total number of non-severe referrals forms | 0 | 10 | 0 | | |
| Mean number of signs and symptoms reported per non-severe referral form | 0.0 | 3.1 | 0.0 | | |
| ***Severe signs symptoms for referral*** |  |  |  | | |
| Illness in child under 2 months old | 0 (0.0) | 0 (0.0) | 0 (0.0) | | |
| Convulsions or fits now or within the past 2 days | 0 (0.0) | 1 (2.9) | 0 (0.0) | | |
| Coma/Loss of consciousness | 1 (5.0) | 0 (0.0) | 0 (0.0) | | |
| Patient is confused or very sleepy - cannot be woken | 1 (5.0) | 0 (0.0) | 0 (0.0) | | |
| Extreme weakness unable to stand or sit without support | 0 (0.0) | 1 (2.9) | 0 (0.0) | | |
| Very hot with temperature of 38.5°C or more | 10 (50.0) | 11 (32.4) | 1 (50.0) | | |
| Very cold with temperature of 35.0°C or less | 0 (0.0) | 0 (0.0) | 0 (0.0) | | |
| Vomiting everything-cannot keep down food or drink | 1 (5.0) | 3 (8.8) | 0 (0.0) | | |
| Not able to drink or breastfeed | 0 (0.0) | 5 (14.7) | 0 (0.0) | | |
| Severe anaemia very pale palms, fingernails, eyelids | 1 (5.0) | 1 (2.9) | 0 (0.0) | | |
| Yellow eyes | 0 (0.0) | 0 (0.0) | 0 (0.0) | | |
| Difficulty in breathing | 5 (25.0) | 8 (23.5) | 1 (50.0) | | |
| Severe dehydration | 0 (0.0) | 1 (2.9) | 0 (0.0) | | |
| Other severe signs and symptoms (free text)^c^ | 1 (5.0) | 3 (8.8) | 0 (0.0) | | |
| **Total number of severe signs and symptoms reported** | **20** | **34** | **2** | | |
| Total number of severe referral forms | 10 | 12 | 1 | | |
| Mean number of signs and symptoms reported per severe referral form | 2.0 | 2.8 | 2.0 | | |
|  |  |  |  | | |
| Total number of severe and non-severe signs and symptoms reported | 20 | 65 | 2 | | |
| Total non-severe and severe referral forms^d^ | 10 | 22 | 1 | | |
| Total referrals made | 186 | 225 | 9 | | |

^a^ Percentages are calculated based on the total number of signs reported.

^b^ Other non-severe signs included: RDT negative; very high temperature but RDT negative (5); doesn't feed well (1), vomiting and loss of appetite (1); RDT positive; cough (1).

^c^ Other severe reasons for referral included: RDT negative; cough and problem with eyes (1), abdominal pain (2); not tested; cough (1);

^d^ 255 referral forms were missing when a referral was made. (71 not tested, 180 RDT-negative and 4 RDT-positive.)

Fig. S1 Job aid for CHWs in a) control (presumptive) arm, b) intervention (RDT) arm


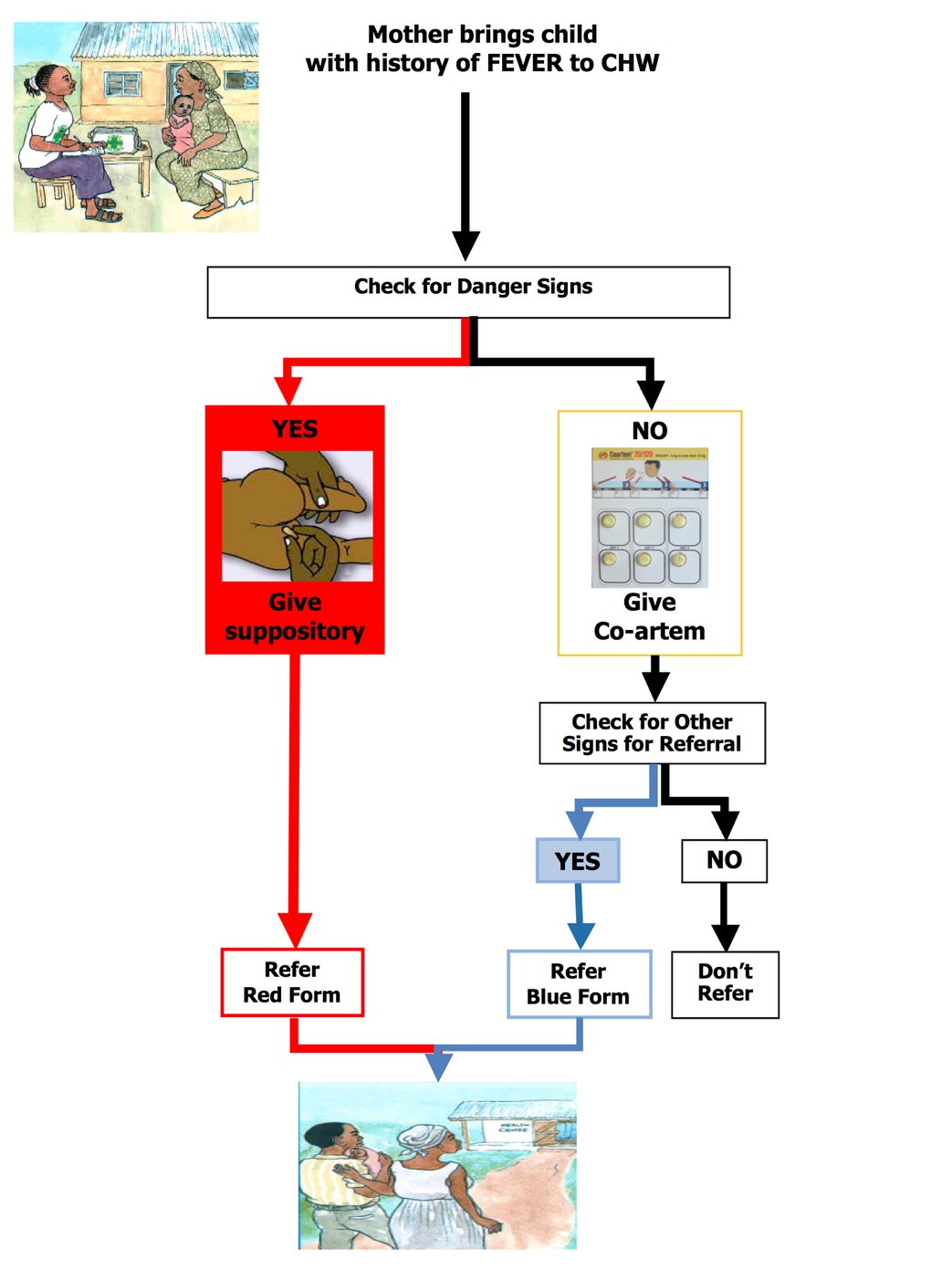

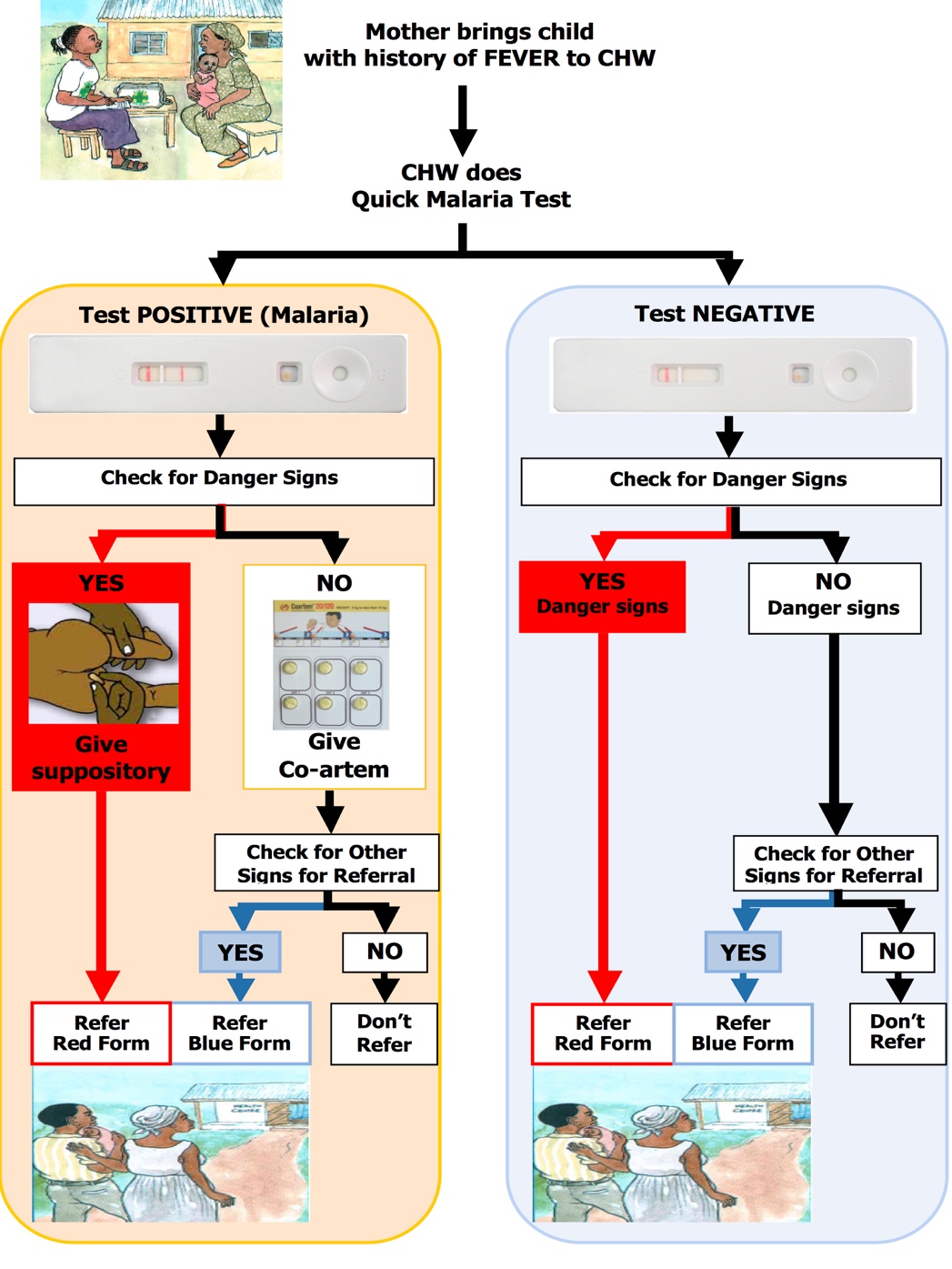


**a) Intervention arm**

**a) Control arm**
